# Special Issue: Monitoring Health Determinants with an Equity Focus

**DOI:** 10.1080/16549716.2017.1410049

**Published:** 2018-01-25

**Authors:** Joy St. John

**Affiliations:** ^a^ Assistant Director-General, Climate and Other Determinants of Health, World Health Organization, Geneva, Switzerland

**Figure:**
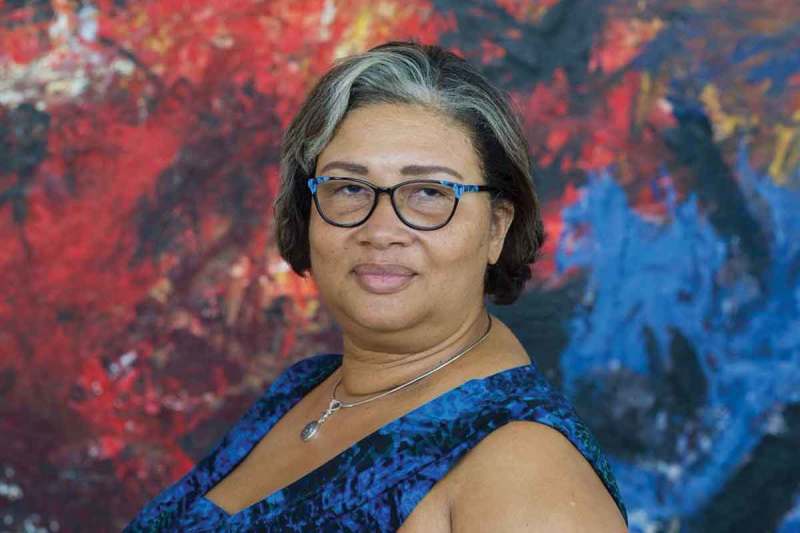

Albert Einstein said that ‘all of science is nothing more than a refinement of everyday thinking.’ In the era of the Sustainable Development Agenda, it is imperative that health tools, services and evidence-based policies address the everyday challenges of sustainability and health. In everyday life, all people, and the more disadvantaged in particular, experience the interconnection between health, climate change and sustainability. Populations in Small Island Developing States, for example, are deprived of livelihoods and increasingly experience devastation from extreme weather; populations in areas of drought and conflict experience soaring food prices; populations in congested and polluted urban areas experience substandard housing conditions, poor air quality or infections from open defecation and poor water and sanitation.

What is measured matters. This is why, at the outset of the Sustainable Development Agenda, strengthening monitoring forms part of WHO’s key strategic measures for addressing the determinants of health and their impacts. Key measures WHO has taken are: (1) to establish that 23% of all global deaths are linked to the environment and 12% are associated with air pollution; (2) to lead development of the measurement of determinants of health and policy interventions, covering, among others, air pollution, energy, water and sanitation, chemicals, radiation, nutrition, children’s environmental health, social determinants, and worker’s health; (3) to establish the Global Coalition for Health, Environment and Climate Change and to participate in cross-governmental partnerships, like the Global Network on Health in All Policies; and (4) to develop instruments that promote understanding of the health impacts across the sustainable development goals, reaching actors within and beyond health, including policy makers and service providers. Key instruments include: the global BreatheLife campaign that works towards a clean air future, policy surveys on financing universal water and sanitation coverage, housing sector guidelines, Health in All Policies trainings, and a chemicals road map for national and international stakeholders.

In line with these achievements, I am delighted to introduce this Special Issue which assembles a number of innovative studies, supported by WHO and a grant from the Rockefeller Foundation. The Special Issue draws attention to indicators and monitoring practices on the determinants of health and their contribution to sustainable development – specifically emphasizing vulnerability, ‘No one left behind’ and social justice. This collection of papers illustrates how the assessment of health determinants points out policies with health gains and other co-benefits. It is essential that WHO makes the health linkages across the sustainable development goals more explicit, reaching all relevant actors. In providing the right evidence, assessments and measures on health determinants at the right time, we can go further, together, in progressing sustainable development.

